# Collaborating With Schools for Public Health Research in England: Lessons Learned for Successful Partnerships

**DOI:** 10.1177/11786302251328831

**Published:** 2025-08-12

**Authors:** Ivelina Tsocheva, Jasmine Chavda, James Scales, Rosamund Dove, Harpal Kalsi, Helen E Wood, Grainne Colligan, Louise Cross, Luke Sartori, Jessica Moon, Aisling Murray, Sarah Van Den Berg, Alice Hirst, Jessica Mitchell, Jason Le, Frances Balkwill, Kristian Petrovic, Esther Lie, Mia Keating, Britzer Vincent Paul Raj, Pavani Kotala, Gurch Randhawa, Ian S Mudway, Chris Griffiths

**Affiliations:** 1 Institute for Health Research, University of Bedfordshire, Luton, UK; 2 Asthma UK Centre for Applied Research, Edinburgh, UK; 3 Wolfson Institute of Population Health, Faculty of Medicine and Dentistry, Queen Mary University of London, UK; 4 Centre of the Cell, Queen Mary University of London, UK; 5 MRC Centre for Environment and Health, Imperial College London, UK; 6 MRC - Asthma UK Centre in Allergic Mechanisms of Asthma, London, UK; 7 NIHR Health Protection Research Unit in Environmental Exposures and Health, Imperial College London, UK

**Keywords:** health assessment, air quality improvement, longitudinal study, school engagement, attrition, recruitment, retention

## Abstract

Carrying out health research with schools can be both challenging and highly rewarding. Here we describe lessons learned from a research partnership lasting over 5 years, initially with 84 primary schools in London and Luton, and extended to 35 secondary schools, during our children health cohort study. This period included school closures and societal disruption during the COVID-19 pandemic, creating additional challenges to ongoing school participation. Our study involved annual health assessment visits to schools to test over 3000 participants and parental self-report questionnaires, to assess the potential benefits of air quality improvements arising from London Ultra Low Emission Zone (introduced in April 2019) on children’s lung development and health. Measures included height, weight, pre- and post- bronchodilator spirometry, physical activity monitoring, cognitive assessment, epigenetic markers of disease risk, SARS-CoV-2 IgE and IgM antibody testing, and heavy metals testing. The average annual participant attrition for our study was 11.6%. The acceptable threshold outlined in the initial protocol was 20%. All schools continued to participate in the study for 5 years. Central to the study success have been: shared agreement on the importance of the research topic; early preparatory work with stakeholders, a parallel engaging and innovative air pollution learning and outreach programme, incentivising school/teacher co-operation and parental questionnaire completion to boost response rates and mitigate non-response bias; and continuity of contact with the accessible and flexible research team. These successes form a template for other health research studies planning long-term engagement with schools.

## Introduction

Meaningful engagement and involvement with participants in public health focused research is widely recognised as being necessary to ensure that research remains relevant, useful and trusted by individuals and communities.^1^ Schools are popular settings for conducting health research involving young people, providing both practical and contextual benefit for health investigators. Working with children in schools is socially significant because findings can have both immediate and long-term benefits in the lives of developing children^2^ and local communities.^3,4^

Schools provide large and often diverse groups of children and young people, conveniently stratified by age, who can be easily approached when successful and trusting relationships have been developed between schools and researchers.^5^ Schools are a safe place for children, where the majority of parents have trust in the organisation and governance. However, trust between community and researchers cannot always be assumed as “one of the major challenges in conducting health research is the understandable lack of trust that often exists between community members and researchers, based on the long history of research that has had no direct benefit. . .and no feedback of the results to the participants involved.”^6^

Furthermore, due to the large number of studies and projects competing for time on a school’s busy schedule, and schools’ overriding priority being the education of their students, many researchers have found it difficult to encourage schools to participate in research projects.^7^ Problems are often related to building relationships and communicating, including ensuring that school partners in research feel heard and valued, managing expectations about project progress, and maintaining continuous participation over time, within the highly dynamic school environment.^8^

It is therefore important for research teams to build positive and effective relationships with school staff and parents to conduct successful research.^5,9^ Research considerations need to be combined with a careful examination of the needs of each individual school in order to build a successful recruitment and retention strategy.^7^ Furthermore, conducting health research with children in schools can be challenging due to: gaining access from gatekeepers, logistics and planning of successful field days, co-operation of school staff, parental consent and maintaining contact and positive relationships, particularly over extended periods.^10,11^ Ongoing engagement is vital for success where studies use designs that require repeated measurements over time, particularly cohort studies.

We describe our approach and insights from our research - a 5-year parallel natural experiment cohort study (initially 4 years but extended by 1 year due the COVID-19 pandemic), working with primary and secondary schools in London and Luton from 2018 to 2023 to test if the introduction of the Ultra Low Emission Zone (ULEZ) in London would produce improved lung growth in children. Sub-studies addressed secondary hypotheses on impacts of the ULEZ on physical exercise, obesity and travel behaviours, and cognitive function development. The study is described in detail elsewhere.^12^

By adopting a school-centred research approach, our research team were able to initially partner with 84 primary schools across the 2 study sites and through these partnerships acquired individual parental consent for participation of 3414 children from school years 2, 3, and 4 (aged 6-9 years) to take part in the study. With a 1-year extension to the data collection period to compensate for school closures during the COVID pandemic, we extended recruitment to 35 secondary schools to which the older children among our cohort transitioned. Our partnerships allowed us to schedule and carry out over 500 visits to schools to collect annual health data from participating children, including: height and weight measurements, spirometry assessments, physical activity and travel behaviours, and in subsets of children: measurements of cognitive function and mental health, capillary blood sampling for COVID antibodies and heavy metals, and saliva sampling for DNA and epigenetic analyses.

School closures and social distancing restrictions during the COVID-19 pandemic, and especially the 3 national lockdowns that took place between March 2020 and March 2021, presented particular challenges in terms of continuing engagement and retention of schools and undertaking data collection. Carrying out research activities in the form in which they were originally planned became impossible, hence the investigators had to find alternative ways in which to continue engaging children, teachers and parents, to continue data collection, and to prevent schools from dropping out.

The aim of this article is to share the experiences and lessons learnt from conducting public health research with children in school settings from our research study before, during and after the COVID-19 pandemic. Our reflections might be beneficial for teams conducting school-based research with children and young people, and for researchers who are exploring paths to broadening research impacts and enhancing outreach to diverse communities. Building and maintaining positive, flexible and effective relationships with school staff and parents, even during challenging times, is vital.^9^

## Methodology

### Research Planning

**Figure 1.**
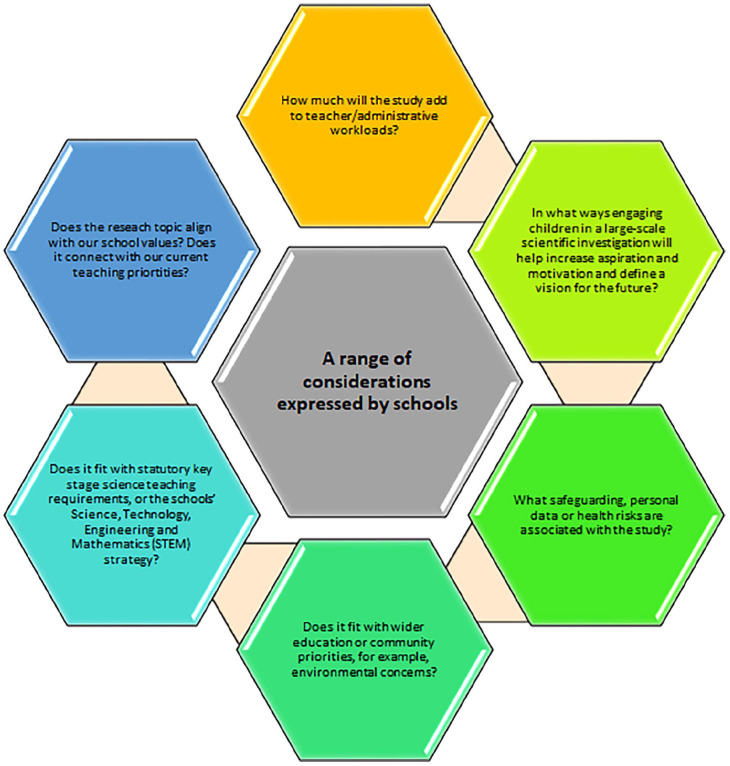
School considerations.

Researchers naturally think their research topic is sufficient of itself to lead to a school’s participation, but our experience shows that schools had a range of different considerations as seen in Figure 1.

Failure to address these fundamental questions, risks recruitment failure at the outset.

In order to mitigate such risk, our team invested time in preparatory work with head teachers, teachers, parents, and children attending schools in the study target area in central London and Luton. We used an informal approach, obtaining permission from head teachers to speak to parents at the school gates of schools eligible for the study, as they collected their children from school. Recent research provided evidence that air pollution levels in Luton was associated with stunted lung growth in primary school children.^13^. We discussed with teachers the optimal means of giving parents information and securing written informed consent (see Attachment 1) for participation – in this case by sending information sheets and consent forms home in the children’s school bags. Every consent form covered all aspects of the study and was signed by the parent and the child.

**Attachment 1.**
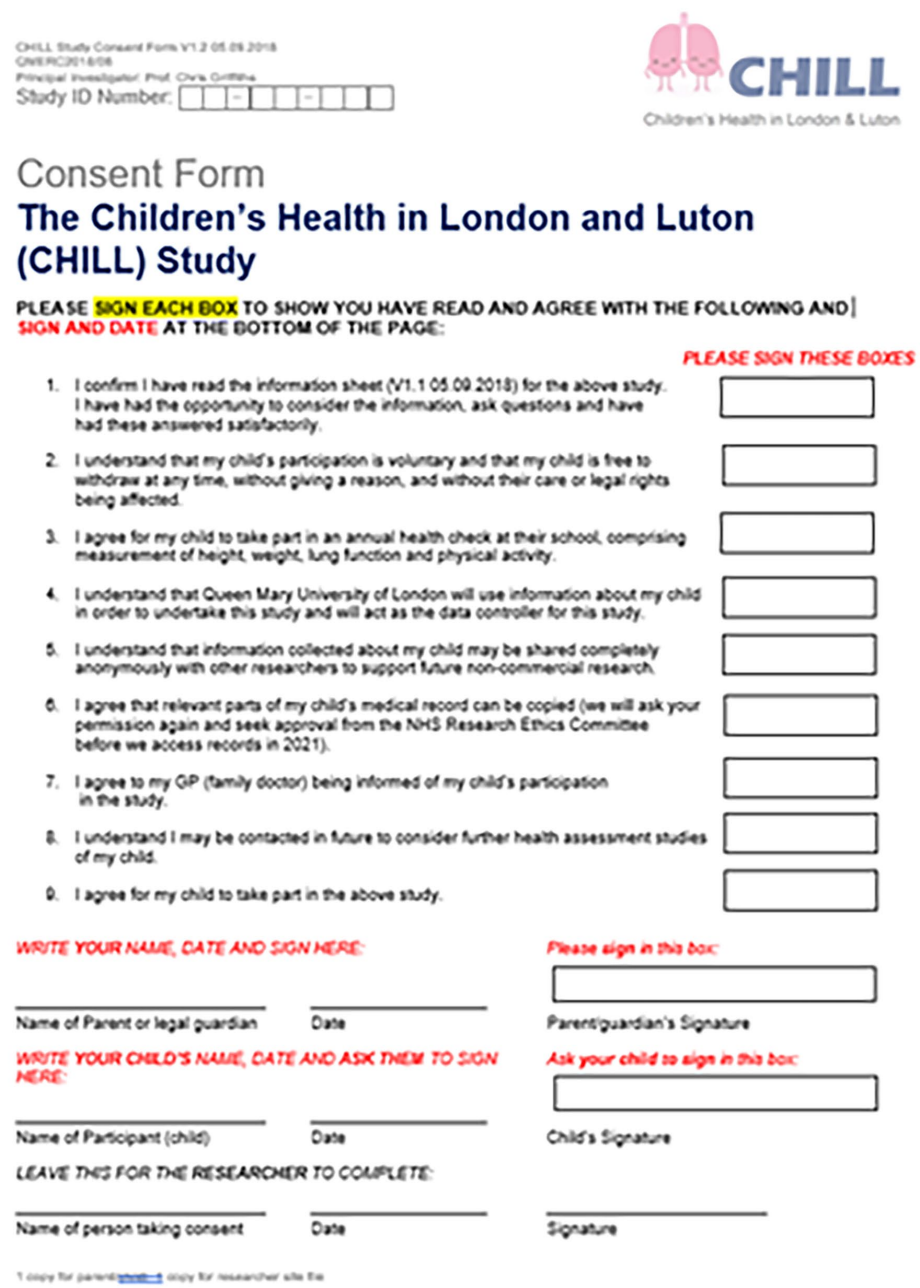
Consent form.

By involving them in the research planning phase, where the research topics and agendas are set, research questions and aims are agreed, and study design and materials are designed, our research study team were able to facilitate their needs.^14^ The stakeholders reflected on their own experiences with air pollution and brought innovative ideas and logistical support to our research study.

### Recruitment of Schools and Children

Considerable data on schools is publicly available on the websites of local authorities and individual schools themselves, enabling researchers to build a picture of the likely numbers of schools and children eligible for a research study.

The 2 pools of schools eligible for recruitment to the study were clearly defined by the study eligibility criteria, in this case state primary schools situated or with catchment areas within the central area of the ULEZ in London (intervention cohort), or within the Borough of Luton (comparison cohort). Schools meeting these criteria were contacted and invited to participate, and meetings arranged with head teachers or their delegates to discuss the study and answer any questions.^12^

Appendix 1 provides an example of our study school Participants Information Sheet.

The results of this school-centred approach enabled us to recruit 84 primary schools and 35 secondary schools’ representative of the range of socioeconomic and ethnic profiles of school-aged children across London and Luton.

Recruitment of the school was the first stage in a partnership, the second stage was recruiting children as participants for the research. Parental consent was sought for each child to participate.

In addition to parent information sheet (Appendix 2) and children information sheet (Appendix 3), the team worked with a professional video company to provide a short engaging YouTube video, involving child actors role-playing the elements of the research. A video was easily accessed via links embedded in emails or QR code scanning and easily watched by parents and children together on mobile phones.

Some of the most useful and practical approaches were found to be school assemblies, playground visits at home time, classroom visits, using the schools’ communication systems and social media.

**Figure 2.**
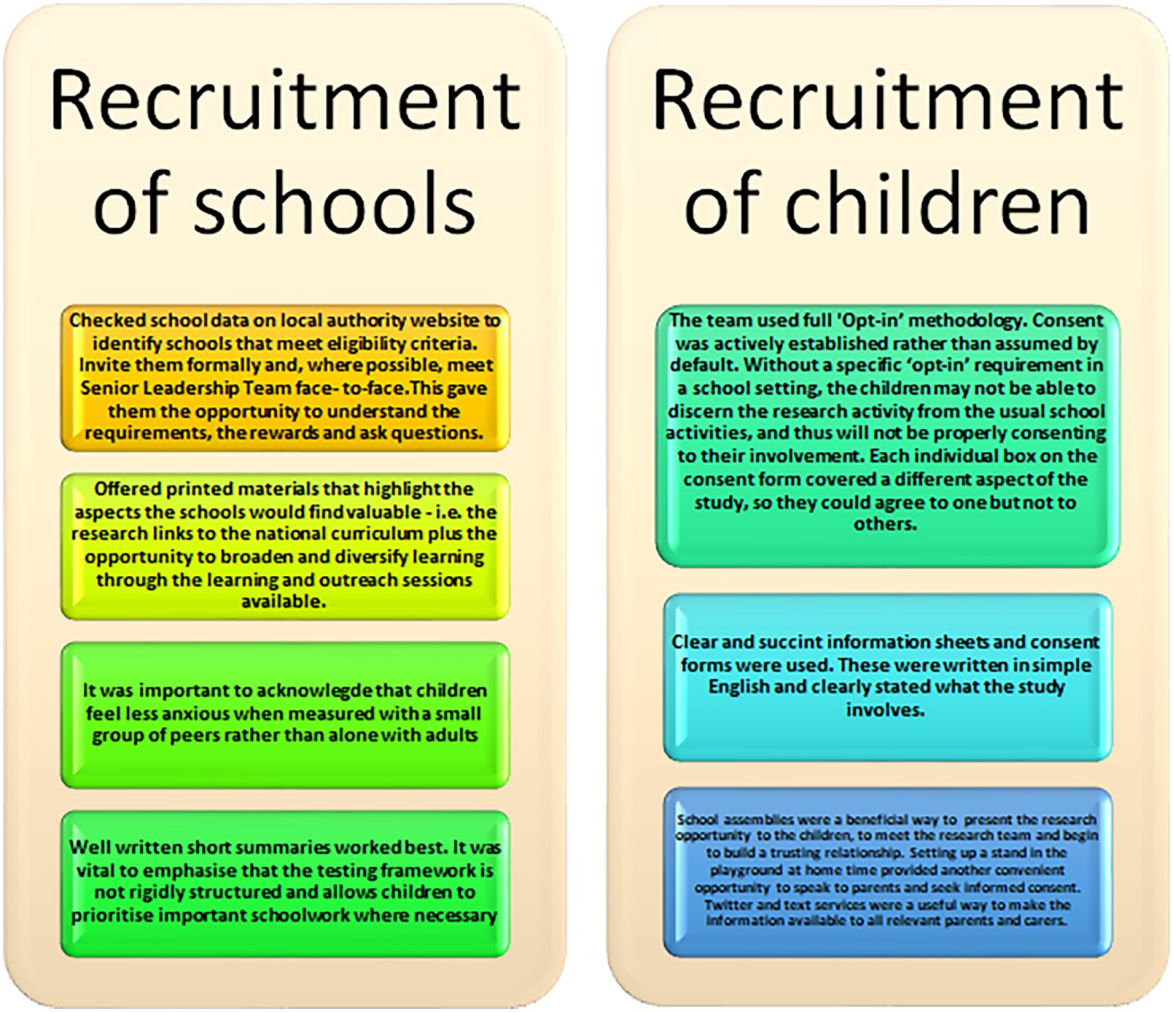
Approaches in Recruitment.

Figure 2 illustrates the approaches that worked for our team during the recruitment process.

### Importance of Public Engagement in Recruitment and Retention

Recruitment of schools and children can also be enhanced through the use of public engagement.^11^ Using local and national media or local radio to increase publicity and the profile of the research can play a vital role in accessing a broader range of families within the local area; this could also include those families who speak English as an additional language, thus increasing interest and subsequently participation in the research Schools and their communities often work very closely together in various aspects of school life - from knowledge and teaching to parental support and empowerment. Therefore, discussions between these communities plays a role in promoting the study and enabling the study to be recognised.

The team organised a high-profile launch for the study and worked closely with contacts in the Public Health units of local authorities to arrange simultaneous school visits. The mayors of London and Luton were present at the launching of the project. These provided ready images and copy for local print, regional and national TV, and social media, leading to widespread dissemination of the study, further promoting the study to other schools in the study areas.

### Ethical and Safe School Visits

Prior to data collection visits, the team ensures an ethical and safe environment for the researchers and the schools involved. Proof of enhanced Disclosure and Barring Service (DBS) checks (as required by the Office for Standards in Education, Children’s Services and Skills (OFSTED) for all study team members visiting schools is a prerequisite for school participation. DBS certificates and work photo IDs were presented upon entry.

### Incentives and Science Outreach

Partnering with schools for research is not only beneficial for the research team, but also for the children involved and their schools. Offering incentives for the school plays an important role in collaboration. Based on feedback from participating schools, the incentives were used to purchase sports equipment, build outdoor learning spaces and fund extra-curricular activities.

Giving the children who participated a small stationery item such as a pen, badge or certificate as a “thank you” is very well-received (Appendix 4), making the children feel valued as part of the research and encourages them to participate again in the future. Parents were also incentivised to return their questionnaires annually. They got a £5 supermarket voucher for returning the health questionnaires.

Offering science educational workshops is a useful way to reach children within the school beyond those consenting to the study. Engaging children in disadvantaged areas in science, especially those under-represented in science, technology and Medicine (STEM) professions including women and those from minoritised ethnic groups is especially important. Children readily understand that their environment is polluted and under threat. Engaging them in interactive informative play readily allows them to understand and begin to articulate views and advocate on this topic.

The team partnered with an award-winning public engagement science group to develop and deliver interactive learning and outreach sessions over the course of the study. These were delivered by a Learning and Outreach Officers. They were STEM subject university graduates who were responsible for the delivery of our interactive learning experiences and building community partnerships. They co-ordinated outreach activities that align with educational standards.

The outreach activities were supported by additional leveraged funding. Topics reflected the additional sub-study elements during the course of the study are outlined in Appendix 5.

These outreach sessions were highly attractive to schools and had an important additional effect: retaining schools and children in the study. Comments were almost invariably positive, including for example, from a teacher: “I’ve never seen my children so engaged in a topic.”

Our learning and outreach extended to the wider community and beyond, for example we delivered sessions at local festivals, national initiatives (MRC event, New Scientist LIVE), and leading science bodies.

### The School Visits

**Figure 3.**
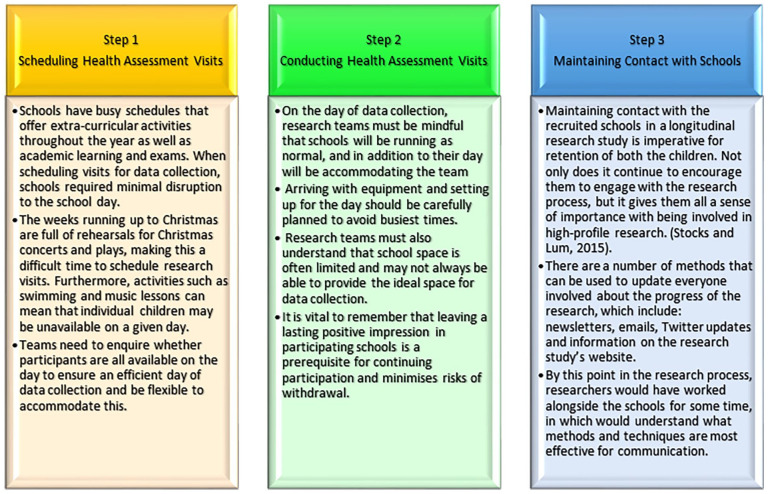
Data collection in 3 steps.

Figure 3 illustrates the data collection in 3 simple steps:

- Step 1. Scheduling Health Assessment Visits
- Step 2. Conducting Health Assessment Visits
- Step 3. Maintaining Contact with Schools

### Sustaining Relationships With Schools and Parents During COVID-19 Pandemic

The COVID-19 pandemic brought unprecedented challenges for society and families. Sudden changes in resources, daily routines and relationships as a result of restrictions on physical interaction resulted in major impacts on families with children. In the absence of school, childcare, extra-curricular activities and family gatherings, children’s social and support networks were severely disrupted. This was particularly evident in families with low levels of social support.^15^

The pandemic presented very specific challenges for our research study. Spirometry (blowing into a machine to measure lung function) is a procedure that can generate an aerosol, presenting a potential cross-infection risk. The team had the double challenge of finding a way of visiting schools in a way that was safe for schools and the study team, but also carrying out spirometry safely.

We identified and worked with 4 “pathfinding” schools who helped us to remain in touch with our participants and research partners. Parents, school staff and children were invited to online meetings that helped the team to identify concerns and priorities by listening to honest, helpful and practical suggestions. They were given the opportunity to raise concerns and voice their questions as active contributors to our revised research strategies. These contacts helped to expand the dialogue and acknowledge lived experience. As research on covid transmission dynamics and risk developed, we found ways of minimising risk by carrying out spirometry assessments outdoors under gazebos, and later, in well-ventilated large rooms, using CO2 monitors to give a proxy of fresh air exchange. These approaches were formally co-designed and risk-assessed with stakeholders and respiratory health experts from European Respiratory Society and Public Health England. The team also created interactive science workshop activities that could be accessed by our study participants in their own time. Questionnaires were sent to participants who we could not visit and without a deadline for completion, thus giving families room and time to adjust to their new routines and roles during lockdown.

### Retention

Various cohort retention and implementation strategies were adopted. Participating schools were given an annual incentive of £250 for each stage of data collection. Staff members with responsibility for participant contact were provided intensive training and support on study protocols, including retention techniques. In recruiting research staff for our study team applicants were screened for experience, communication skills, cultural-competence, and specialised knowledge of the population (ie, in Luton where a large part of the cohort was of Asian/Asian British origin, researchers with similar ethnic backgrounds were employed. A number of different contact details for participants were recorded and these updated at every participant contact.

20 secondary schools in London with small numbers (<5 children per school) of our cohort participants were identified; The team did not have the resources to visit these schools to carry out health assessments. To retain and gather data on these participants, we ran a health assessment event in central London on 2 consecutive study years (Y4 and Y5). These combined health assessments with science engagement sessions. Centralised testing flyers were sent to participants (Appendix 6). These proved popular and successful and were attended by over 70 children.

Cohort retention issues were discussed through meetings involving research assistants, project managers and principal investigators. During these meetings, the study team examined the latest recruitment and retention rates and discussed strategies and ideas for participants who were difficult to contact.

Table 1 illustrates the challenges encountered by the research team and the measures taken to resolve them.

**Table 1.** Research tasks and challenges, measures and outcomes.

Research tasks and challenges	Measures taken	Outcomes
Preparatory work with stakeholders across both sites	• In London, the team visited schools, talked to school staff and parents about air pollution and its impact on our health • Sought feedback from environmental campaign groups in London • In Luton, the team liaised with the Public Health, Commissioning & Procurement department at the local council prior to launching the study • Got feedback on how best to run the assessment and input on our protocol across both sites • Piloted parent questionnaires • Had a STUDY launch event with mayors in London and Luton covered by media • In London, the team took part in local public health forums such as Love Luton: Investing in success, investing in health, and “No education without health education” Primary and Secondary School Health & Well-being Conference.	• Parents across both sites were willing to engage in a discussion about air quality but the problem was more highly publicised in London • In Luton, parents lacked specific knowledge about the Ultra Low Emission Zone (ULEZ) implementation in London, but they expressed concerns about traffic volume from the motorway, local traffic restrictions and the installation of electric vehicle rapid charging units and Gas-To-Liquid refuelling infrastructure • Managed to adjust protocol accordingly based on feedback • As a result of media coverage, the study’s profile was raised and key opportunities for co-operation with schools in target areas were identified
Initial recruitment of primary schools in target areas	• Approached all schools that meet selection criteria • Engaged Head teachers, organised meetings with Senior Leadership to answer any questions • Offered each school an annual incentive for taking part	• 67% of primary schools approached in London and 69% of schools approached in Luton signed up for the study • The schools that declined to sign up for STUDY or did not respond to our contact attempts did not give children from certain neighbourhoods the opportunity to take part in the study
Participant recruitment in busy schools	• Engaged non-teaching staff • Prepared concise materials in simple English to present at school assemblies and used visuals (pictures and videos whenever possible) • Arranged brief classroom visits to engage children • Spoke to parents in school playground at home time	• Recruited over 3414 children across London and Luton and exceeded our recruitment target of 3120 by over 8%. • Some recruitment strategies worked better in London (i.e. classroom visits because the schools were smaller), whereas others worked better in Luton (i.e. large assemblies in the school hall because schools had 4 or 5 classes per school year)
Engaging parents from diverse populations and with different levels of literacy 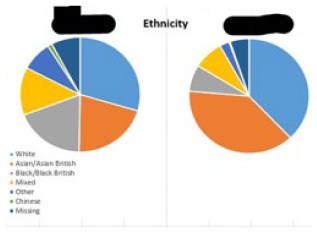 Image 1. Ethnicity distribution across London and Luton	• If the child collecting parent spoke limited English, co-operation was sought from other family members • Where possible help from family workers and diversity and inclusion officers was sought as they usually spoke the languages of local parents • In Luton, the team used Urdu and Bengali speaking student helpers from the local university to engage with parents	• Recruited an ethnically diverse cohort. 62.6% of participants in London and 56.9% of participants in Luton were from BAME backgrounds • Different ethnicity distribution across both sites • Using paper parent questionnaires allowed the team to include parents with low digital literacy • Had lower parent questionnaire return rates from people who spoke limited English
Educate, engage and inspire children from disadvantaged backgrounds	• Explained to children how testing equipment works, what the results show and gave them tasks, not instructions • Careful consideration of contexts and use of child-centred research skills and methods • Interactive science outreach sessions covering study topics were made available to all children in the targeted school years • Children incentives were given for taking part – certificates, pens, pencils and badges branded with the research logo (Appendix 4)	• Successful post-bronchodilator spirometry was achieved with 80% of participating children • Due to evolving communication skills and different social and cognitive capacities, some children needed more time to process instructions or were unable to complete the spirometry manoeuvres • Delivered science outreach sessions to over 11 000 children over a 5-y period. • Schools reported increased interest in STEM subjects as a result of the study team interacting with participating children
Resuming data collection visits during and after the pandemic	• In the beginning, outdoor testing was employed • As the restrictions were relaxed, Perspex partitions were used to create a physical barrier between the researcher and the participating child. • Researchers wore suitable PPE (disposable gloves, aprons and FFP2 masks). • Lung function testing was done with bacterial viral filter (over 99.99% effective in preventing bacterial and viral transfer). • Salbutamol (blue inhaler) was administered using disposable spacers (one per child). • All equipment was cleaned according to manufacturer guidelines between each child. • C02 monitors were used	• These multiple adjustments made to the study SOP allowed the team to return to data collection duties soon after the last lockdown • Co-operation from pathfinding schools across both sites helped to put new implementations in practice • Had to account for various forms of restrictions and tiered systems with varied local modifications across London and Luton • Due to regional differences across both sites, there were some schools that remained closed to visitors throughout the school year 2020 to 21 and only let the study team back in from the start of the new school year in September 2021
Recruiting secondary schools after the pandemic to collect data from traced participants	• Performed tracing activities to identify which secondary schools participants have gone to • Engaged Head teachers, organised meetings with Senior Leadership to answer any questions • After tracing, prompts were sent out to recruited schools to identify participants who were not traced yet • In London, children were distributed across a much larger number of secondary schools, therefore a Centralised Testing event was scheduled for Study Years 4 and 5	• About 60% of participants were successfully traced into secondary schools • The participants who were not traced were lost to follow up due to non-contact, incorrect contact details or moving out of study area • Encountered difficulties when trying to engage secondary schools at first, as they were not familiar with the study • Secondary schools were much larger and teachers were not easily contactable • Secondary schools had limited room availability and time constraints, so the researchers had to work flexibly
Mitigating school/participants withdrawal risk and keeping attrition levels low, including during and after the pandemic	• School incentive was conditional and paid in annual instalments after data collection visits. School certificate was also given • Low value (£5 Sainsbury’s voucher) was given for parent questionnaire completion • Hosted a study update zoom workshop for children and parents during lockdown • Kept children engaged via webinars and online activities • Record as many contact details as possible to perform tracing activities if necessary	• No school has dropped out of the study across a 5-y period • Adding a fifth year to the study due to the pandemic heightened the participant attrition risk • Despite adding a fifth year and going through a global pandemic, the attrition rates remained lower than the 20% annual attrition expected in the initial protocol • People moved out of central London during and after the pandemic, resulting in some participant loss to follow up • Over time, a drop-off of parent questionnaire return was observed

## Discussion

Recruiting and retaining participants in health research is often challenging, especially in ethnically and culturally diverse populations living in disadvantaged communities. These communities face unique challenges, adversities and inequalities.^16^ We presented practical solutions that have helped to recruit and retain schools and participants over a 5-year period, as an exemplar for future longitudinal studies in school-aged children.

Engaging everyone involved in the research (school staff, parents, children, local council members etc.) at the earliest possible opportunity, helped the research team to make informed decisions about the research process and make some practical changes. Benefits included getting input on research activities, developing culturally sensitive approaches and enhancing the recruitment and retention of research participants.^17^ Stakeholder engagement is a powerful vehicle for effectuating changes that can improve health.^18^. Engaging community health stakeholders in the research process is often the missing link to improving the quality and outcomes of health promotion activities, disease prevention initiatives, and research studies.^19^

Maintaining stakeholder engagement for the full duration of the study is a long-term process that requires time and effort from all sides. It builds trust, values all stakeholders’ contributions, and generates a collaborative framework.^20^

Children and young people are viewed as active contributors in the project, rather than objects of research.^21^ Adopting involvement strategies such as learning and outreach sessions, that play to children’s competencies and strengths is vital. Engagement of children and young people requires the use of creative, participatory methods, tools and involvement techniques to reveal children’s abilities. Participating children learned about lung function by exploring lung props, making slime, building green walls with plants, drawing with charcoal and performing breathing exercises with straws and feathers. Attachment 2 is an excerpt of our workshop brochures. Research is often planned and described in emotionally “neutral” terms, although participatory research necessarily relies on building relationships and engaging emotionally in a research process with others.^22^ Embedding the enjoyment of interactive learning about the main topic of the study within the participatory research process, was an essential part of the project. Learning and outreach sessions for the children on the study health assessment days were a major component of our recruitment and retention strategy, reinforcing the positive relationships between researchers, children, and the schools. Although many funders do not see this as a fundamental part of research which often results less financial support, it plays a key role in the study experience. Children are inquisitive by nature, therefore introducing research and workshop-based activities into their typical school day is exciting for them and makes full use of their inquisitive nature. Curiosity is a powerful driver of learning.^23^ Additionally, in educational settings, curiosity for scientific knowledge is a major motivation for long-term involvement in STEM subjects and predicts academic performance.^24,25^ Furthermore, science education policy documents worldwide highlight the importance of scientific literacy and suggest it is essential that pupils develop their understanding about the processes of science and the type of knowledge science produces and have the ability to apply this in everyday contexts.^26^

**Attachment 2.**
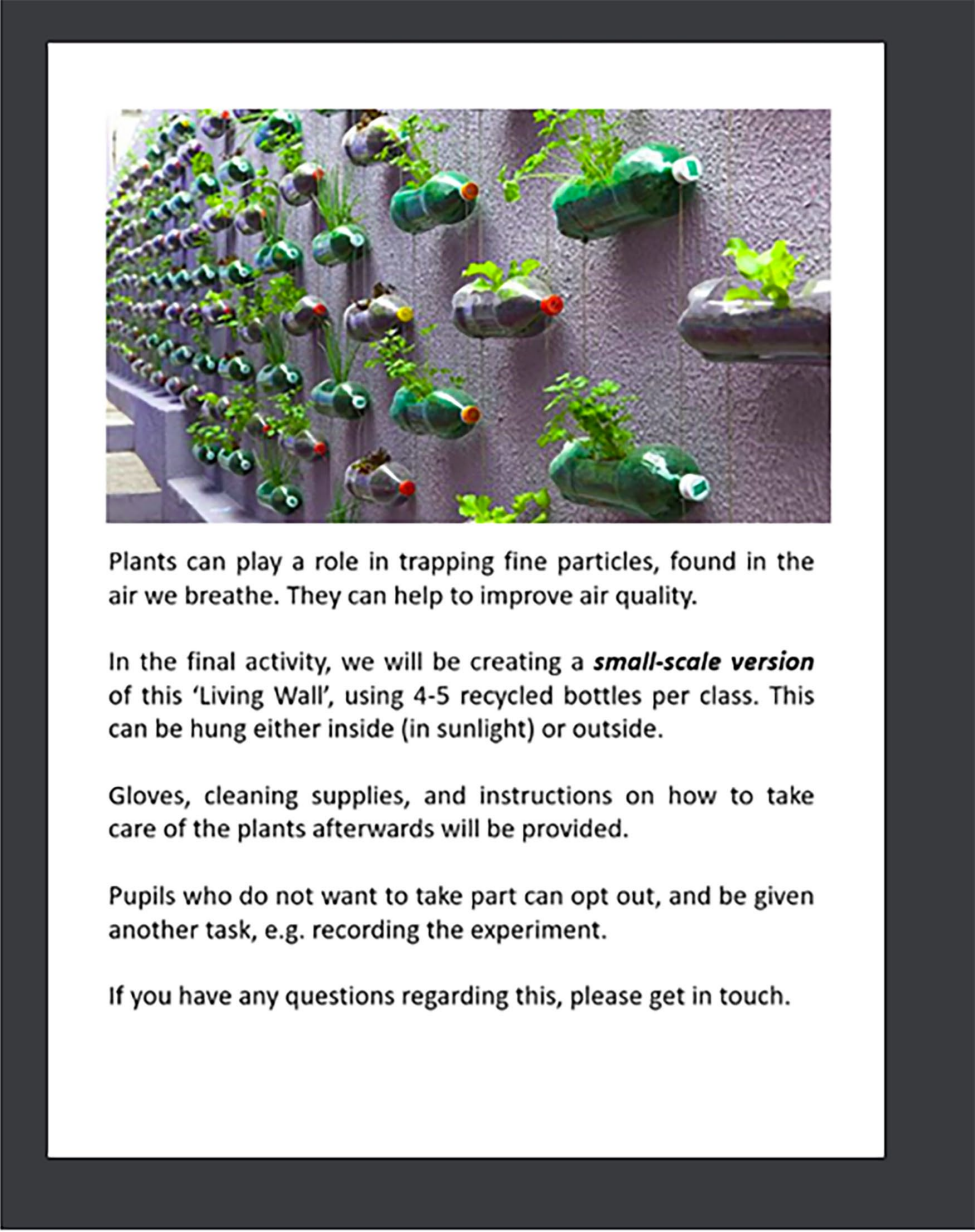
“Pollution solution” workshop activity.

One of the often-cited criticisms of health research is its lack of pragmatism, frequently addressing questions and outcomes of limited relevance to the end-users. This means a significant proportion of health research is potentially wasted from the outset, because researchers have not consulted with end users when prioritising an area of research or selecting a specific research question.^27^ Building trust between investigators and community members is vital and could enhance protocol development and implementation.^28,29^ Engaging the community can improve the quality, validity, and relevance of research findings, and it can empower community members to advocate for programme and policy changes that may be indicated as a result of the research.^17^

### Implications for Practice

The lessons learned from our research team during the 5-year study period appear to have enabled us to maintain 84 primary schools’ interests, expand successfully into 35 secondary schools and keep all schools on board for the whole study, including during the pandemic. The team’s tailored approach, flexibility and engagement have been vital to secure their continuous engagement with the project. Our learning journey could enable more clinical researchers to study children’s health in school settings. The combination of recruitment and retention strategies outlined in this manuscript form a working template for other health studies planning long-term engagement with schools.

### Implications for Policy

The tangible benefits from this study include: (A) Creating valuable educational and health promotion opportunities in areas of high deprivation where fewer resources and opportunities are available, (B) Inform policy discussion and improve decisions leading to better air quality, health, and wellbeing. Our efforts align with the Framework for Action developed by WHO that aims to enhance the ongoing work in the health and education sectors and to contribute to the achievement of other global commitments for child health. Successful partnerships in health research offer schools, parents, teachers, communities, and other stakeholders opportunities to build healthier lives and add to the creation of global standards for health promoting schools.

## References

[1] Macaulay AC. Participatory research: what is the history? Has the purpose changed? Fam Pract. 2017;34(3):256-258. 27993909 10.1093/fampra/cmw117

[2] Vukotich CJ Cousins J Stebbins S. Building sustainable research engagements: lessons learned from research with schools. J Res Pr. 2014;10(1):M1.

[3] O’Mara-Eves A Brunton G McDaid D , et al. Community engagement to reduce inequalities in health: a systematic review, meta-analysis and economic analysis. Southampton (UK): NIHR Journals Library; 2013 November. Accessed October 4, 2023. (Public Health Research, No. 1.4.). https://www.ncbi.nlm.nih.gov/books/NBK262817/25642563

[4] Thomson LJ Gordon-Nesbitt R Elsden E Chatterjee HJ. The role of cultural, community and natural assets in addressing societal and structural health inequalities in the UK: future research priorities. Int J Equity Health. 2021;20:249. 34819080 10.1186/s12939-021-01590-4 PMC8611639

[5] Bruzzese JM Gallagher R McCann-Doyle S Reiss PT Wijetunga NA. Effective methods to improve recruitment and retention in school-based substance use prevention studies. J Sch Health. 2009;79:400-407. 19691714 10.1111/j.1746-1561.2009.00427.x

[6] Israel BA Schulz AJ Parker EA Becker AB. Community-based participatory research: policy recommendations for promoting a partnership approach in health research. Educ Heal. 2001;14(2):182-197. 10.1080/13576280110051055 14742017

[7] Midford R McBride N Farrington F. Conducting research in schools: lessons learnt from experience. Health Promot J Austr. 2000;10(1):41-68. Official Journal of Australian Association of Health Promotion Professionals.

[8] Shen S Doyle-Thomas KAR Beesley L , et al. How and why should we engage parents as co-researchers in health research? A scoping review of current practices. Health Expect. 2017;20(4):543-554. 27516003 10.1111/hex.12490 PMC5513005

[9] Bartlett R Wright T Olarinde T , et al. Schools as sites for recruiting participants and implementing research. J Community Health Nurs. 2017;34(2):80-88. 28467204 10.1080/07370016.2017.1304146 PMC6080250

[10] Neuman G Shavit I Matsui D Koren G. Ethics of research in pediatric emergency medicine. Paediatr Drugs. 2014;17:69-76. 10.1007/s40272-014-0110-4 25475848

[11] Stocks J Lum S. Back to school: challenges and rewards of engaging young children in scientific research. Arch Dis Child. 2015;101(9):785-787. 10.1136/archdischild-2015-310347 PMC5013085 27117837

[12] Tsocheva I Scales J Dove R , et al. Investigating the impact of London’s ultra low emission zone on children’s health: children’s health in London and Luton (CHILL) protocol for a prospective parallel cohort study. BMC Pediatr. 2023;23:556. 37925402 10.1186/s12887-023-04384-5 PMC10625305

[13] Mudway IS Dundas I Wood HE , et al. Impact of City 1’s low emission zone on air quality and children’s respiratory health: a sequential annual cross-sectional study. Lancet Public Health. 2018;4:e28-e40. 10.1016/S2468-2667(18)30202-0 PMC6323357 30448150

[14] Watkins BX Shepard PM Corbin-Mark CD. Completing the circle: a model for effective community review of environmental health research. Am J Public Health. 2009;99:S567-S577. 10.2105/AJPH.2008.149369 PMC2774186 19890159

[15] Brown SM Doom JR Lechuga-Peña S Watamura SE Koppels T. Stress and parenting during the global COVID-19 pandemic. Child Abuse Negl. 2020;1:104699. 10.1016/j.chiabu.2020.104699 PMC7440155 32859394

[16] Grape A Rhee H Wicks M Tumiel-Berhalter L Sloand E. Recruitment and retention strategies for an urban adolescent study: lessons learned from a multi-center study of community-based asthma self-management intervention for adolescents. J Adolesc. 2018;65:123-132. 29587184 10.1016/j.adolescence.2018.03.004 PMC5932256

[17] Israel BA Schulz AJ Parker EA Becker AB. Review of community-based research: assessing partnership approaches to improve public health. Annu Rev Public Health. 1998;19:173-194. 9611617 10.1146/annurev.publhealth.19.1.173

[18] Fawcett SB Paine-Andrews A Francisco VT , et al. Using empowerment theory in collaborative partnerships for community health and development. Am J Community Psychol. 1995;23(5):677-697. 8851345 10.1007/BF02506987

[19] Minkler M. Ethical challenges for the “outside” researcher in community-based participatory research. Health Educ Behav. 2004;31(6):684-697. 15539542 10.1177/1090198104269566

[20] Butterfoss FD Francisco VT. Evaluating community partnerships and coalitions with practitioners in mind. Health Promot Pract. 2004;5(2):108-114. 15090164 10.1177/1524839903260844

[21] Kennan D Horgan D . Contribution of participatory research with children and young people to policy. In: Coyne I Carter B , eds. Being Participatory: Researching With Children and Young People. Springer; 2024:65-83.

[22] Wright LHV Tisdall K Moore N . Taking emotions seriously: fun and pride in participatory research. Emot Space Soc. 2021;41:100836.

[23] Liquin EG Lombrozo T. Explanation-seeking curiosity in childhood. Curr Opin Behav Sci. 2020;35:14-20.

[24] Levrini O De Ambrosis A Hemmer S , et al. Understanding first-year students’ curiosity and interest about physics—lessons learned from the HOPE project. Eur J Phys. 2016;38:2016.

[25] von Stumm S Hell B Chamorro-Premuzic T . The hungry mind: intellectual curiosity is the third pillar of academic performance. Perspect Psychol Sci. 2011;6:574-588. 26168378 10.1177/1745691611421204

[26] Eurydice Network. Science Education in Europe: National Policies, Practices and Research. Education. Audiovisual and Culture Executive Agency; 2011.

[27] Ioannidis JPA . Why most clinical research is not useful. PLoS Med. 2016;13:e1002049. 10.1371/journal.pmed.1002049 PMC4915619 27328301

[28] O’Fallon LR Dearry A. Community-based participatory research as a tool to advance environmental health sciences. Environ Health Perspect. 2002;110(suppl 2):155-159. 11929724 10.1289/ehp.02110s2155 PMC1241159

[29] Dickert N Sugarman J. Ethical goals of community consultation in research. Am J Public Health. 2005;95:1123-1127. 15983268 10.2105/AJPH.2004.058933 PMC1449329

